# FGCD: a database of fungal gene clusters related to secondary metabolism

**DOI:** 10.1093/database/baae011

**Published:** 2024-03-19

**Authors:** Fuyuan Zhang, Hongzhe Cao, Helong Si, Jinping Zang, Jingao Dong, Jihong Xing, Kang Zhang

**Affiliations:** State Key Laboratory of North China Crop Improvement and Regulation, Hebei Agricultural University, No. 289 Lingyusi Street, Baoding 071000, China; College of Life Science, Hebei Agricultural University, No. 289 Lingyusi Street, Baoding 071000, China; College of Life Science, Hebei Agricultural University, No. 289 Lingyusi Street, Baoding 071000, China; Hebei Key Laboratory of Plant Physiology and Molecular Pathology, Hebei Agricultural University, No. 289 Lingyusi Street, Baoding 071000, China; College of Life Science, Hebei Agricultural University, No. 289 Lingyusi Street, Baoding 071000, China; Hebei Key Laboratory of Plant Physiology and Molecular Pathology, Hebei Agricultural University, No. 289 Lingyusi Street, Baoding 071000, China; Hebei Key Laboratory of Plant Physiology and Molecular Pathology, Hebei Agricultural University, No. 289 Lingyusi Street, Baoding 071000, China; State Key Laboratory of North China Crop Improvement and Regulation, Hebei Agricultural University, No. 289 Lingyusi Street, Baoding 071000, China; Hebei Key Laboratory of Plant Physiology and Molecular Pathology, Hebei Agricultural University, No. 289 Lingyusi Street, Baoding 071000, China; State Key Laboratory of North China Crop Improvement and Regulation, Hebei Agricultural University, No. 289 Lingyusi Street, Baoding 071000, China; College of Life Science, Hebei Agricultural University, No. 289 Lingyusi Street, Baoding 071000, China; State Key Laboratory of North China Crop Improvement and Regulation, Hebei Agricultural University, No. 289 Lingyusi Street, Baoding 071000, China; College of Life Science, Hebei Agricultural University, No. 289 Lingyusi Street, Baoding 071000, China; Hebei Key Laboratory of Plant Physiology and Molecular Pathology, Hebei Agricultural University, No. 289 Lingyusi Street, Baoding 071000, China

## Abstract

Fungal secondary metabolites are not necessary for growth, but they are important for fungal metabolism and ecology because they provide selective advantages for competition, survival and interactions with the environment. These various metabolites are widely used as medicinal precursors and insecticides. Secondary metabolism genes are commonly arranged in clusters along chromosomes, which allow for the coordinate control of complete pathways. In this study, we created the Fungal Gene Cluster Database to store, retrieve, and visualize secondary metabolite gene cluster information across fungal species. The database was created by merging data from RNA sequencing, Basic Local Alignment Search Tool, genome browser, enrichment analysis and the R Shiny web framework to visualize and query putative gene clusters. This database facilitated the rapid and thorough examination of significant gene clusters across fungal species by detecting, defining and graphically displaying the architecture, organization and expression patterns of secondary metabolite gene clusters. In general, this genomic resource makes use of the tremendous chemical variety of the products of these ecologically and biotechnologically significant gene clusters to our further understanding of fungal secondary metabolism.

**Database URL**: https://www.hebaubioinformatics.cn/FungalGeneCluster/

## Introduction

Primary and secondary metabolites are the two types of metabolite produced by living organisms, particularly plants and microorganisms Microbial primary metabolism refers to the basic but essential metabolic processes that microorganisms need to generate or release energy, acquire nutrients and assemble the core components needed for continued growth and replication (1). Microbial primary metabolism involves, among other things, the synthesis of fatty acids, amino acids, nucleic acids, the pentose phosphate route, the tricarboxylic acid cycle and glycolysis. Through these metabolic pathways, microorganisms are able to absorb and digest nutrients from their surroundings, use energy and create the fundamental biomolecules required to build new cells. Since primary metabolic pathways facilitate vital biological functions common to most microbial species, they are highly conserved.

On the other hand, secondary metabolism (SM) involves pathways that are involved in the adaptation, competition and interaction of microorganisms with their particular environment, but which are not essential for regular cell function and growth. More species-specific roles for secondary metabolites include pathogenicity, defense, signaling and stress response. SM pathways allow for specialized microbial activities, whereas primary metabolic processes are centered on microbial survival. Secondary metabolites, however, are often specific to individual microbial species and culture conditions, being found in only a limited number of microorganisms (2). Nonetheless, the literature indicates that certain fungal secondary metabolites can protect fungi from abiotic, e.g. UV, and biotic stresses, e.g. bacteria, while encouraging the growth of fungi. From an applied viewpoint, some fungal secondary metabolites are used to manage insect pests and crop weeds (3).

Fungal secondary metabolite biosynthesis genes are typically grouped together on chromosomes to create gene clusters, which encode functionally related enzymes that catalyze the various steps in the biosynthesis of certain chemicals (4). Up to 40 gene clusters encoding the production of secondary metabolites have been identified in microbial genomes. One or more transcription products are formed by each gene in the gene cluster. Gene clusters typically contain one or more gene types, including defense genes, regulatory genes, transporter-coding genes and structural genes. As long as one of these genes can be retrieved, the full gene cluster sequence can be cloned because these genes are located close to each other in the gene cluster. The literature reports that this feature has been used to clone over 40 fungal secondary metabolite gene clusters and has also shown the relationship between secondary metabolites and various gene systems (5). An SM biosynthesis gene cluster, however, often consists of three or more non-homologous genes. These non-homologous genes include the ones, which encode structural genes and which encode enzymes, that create the carbon skeleton of secondary metabolites and modification enzymes that are employed to alter the carbon skeleton of metabolites. Furthermore, certain genes play roles in the synthesis of enzymes, which, via splicing, transcripts enzyme-encoding genes around them (6).

Functionally co-regulated clusters of the expression of fungal secondary metabolite genes involve several aspects. Several resistance genes have been identified in the majority of fungal secondary metabolite biosynthetic gene clusters (BGCs), and resistance and structural genes are coordinately regulated, leading to either synergistic or antagonistic effects. The majority of the genes within the gene cluster are co-expressed, encoding the metabolic enzymes to function in a common pathway under particular conditions or at specific stages (7). Occasionally, however, there are isolated genes within a gene cluster that are transcribed separately. Transcription plays a major role in the regulation of expression of gene clusters associated with fungal secondary metabolite biosynthesis. Transcriptional regulation is influenced by global regulatory factors of other intracellular material creation and cell differentiation, in addition to specific regulatory factors, such as transcription factors, controlling the expression of certain pathways (8).

Fungal secondary metabolite mining is currently one of the most intriguing areas of microbial science. The genomes of a growing number of filamentous fungi have been fully sequenced and assembled as a result of advances in sequencing technology and decreasing sequencing costs, and studies have shown that the size of the entire genome sequence of a filamentous fungal species is positively correlated with the number of BGCs present. This finding suggests that the genes that contribute to the biosynthesis of secondary metabolites in fungi have not yet been thoroughly dissected.

**Figure 1.**
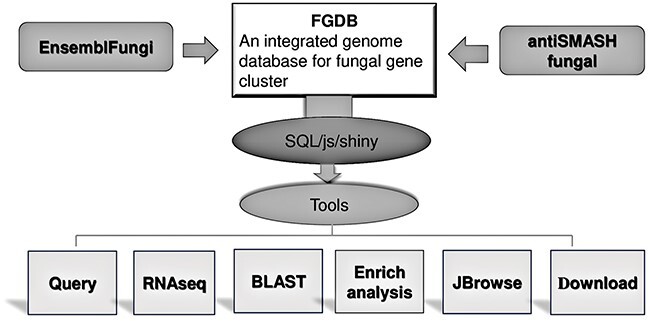
FGCD database structure and function It includes the composition of the database as well as the data sources and functions provided.

Using a range of bioinformatic analysis methods, the Fungal Gene Cluster Database (FGCD) was constructed. This database could be used to query fungal gene clusters for pertinent data. The tool simultaneously offers genome browsing, enrichment analysis based on gene groups and Basic Local Alignment Search Tool (BLAST). There are now 241 filamentous fungal species represented in the FGCD database, comprising 7597 SM BGCs and containing 97 159 genes overall (Figure 1), which provide a useful platform for functional annotation and enrichment analysis of fungal SM biosynthetic genes.

## Materials and methods

### Data collection

By collecting pertinent genomic data from the Ensemble Fungi database (http://fungi.ensembl.org/index.html), we assembled a large dataset of 241 fungal species. For individual species, the database included protein sequences, coding DNA sequences, full genome sequences and genome annotations; RNA-Seq datasets of 70 fungal species were collected from the National Center for Biotechnology Information (NCBI) database (9). The RNA-Seq (RNA sequencing) data included a range of situations, such as diverse tissue types, and stages of growth and development. To measure gene expression, the raw RNA-Seq data were subjected to normal bioinformatic pipeline analysis. Subsequently, the gene expression data were examined, especially for genes within anticipated secondary metabolite clusters, using unique Python programs developed in-house. In this way, it is possible for the database to combine transcriptomic data that match genomic cluster data for thorough analysis. The mapping of cluster gene expression using the RNA-Seq dataset will be extended over time to include other species, environments and tissue types. The combination of transcriptomic, gene cluster and genomic data from open sources offers a wealth of multi-omics information about fungal SM.

### Identification and data integration of fungal gene clusters

The distinctive sequence structure and gene arrangement of secondary metabolite genes have been leveraged to develop specialized software for their analysis. Annotation methods for gene clusters involved in secondary metabolite biosynthesis are expanding in tandem with the rapid growth of sequencing data. To annotate fungal secondary metabolite gene clusters, we employed antibiotics & Secondary Metabolite Analysis Shell (antiSMASH) (https://antismash.secondarymetabolites.org/). Using a range of bioinformatic analytical methods, antiSMASH is a tool that combines a hidden Markov model to find all of the gene clusters involved in secondary metabolite biosynthesis in the archaeal, bacterial and fungal genomes and predict secondary metabolites (10). Using the core biosynthetic genes as a guide, antiSMASH can determine the synthesis pathways of 45 secondary metabolites based on the clustering algorithm. For some of the more intricate secondary metabolites, including thiopeptides, terpenoids and polyketides, more precise predictions of their BGCs can be calculated with antiSMASH (11). Then, using its own Python and R scripts developed in-house, the antiSMASH analysis results are incorporated into the SQLite database engine.

### Collection and collation of RNA-Seq data

We obtained the RNA-Seq datasets of 70 fungal species from the NCBI, which were primarily related to growth, infection and various tissues. RNA-Seq was used to evaluate the original data, which were retrieved from NCBI. Additional analysis was carried out using the information on the projected gene cluster. We extracted the analysis data pertaining to gene clusters using our custom Python script. Simultaneously, utilizing the obtained gene cluster information as background, the enrichment analysis based on the gene cluster background is introduced. Finally, all data were integrated into the FGCD database and online services were rendered using the R ‘Shiny’ package, with which RStudio (https://posit.co/) was used to create the web page framework. Users need have no experience with HTML, CSS or JavaScript to use ‘Shiny’ to develop a network application framework that allows for quick two-way communication between a browser and R (12). Although it is highly compatible with HTML and CSS, using the ‘Shiny’ package to create interactive web apps does not require much front-end experience (13).

## Results

### Fungal gene cluster data query

**Figure 2.**
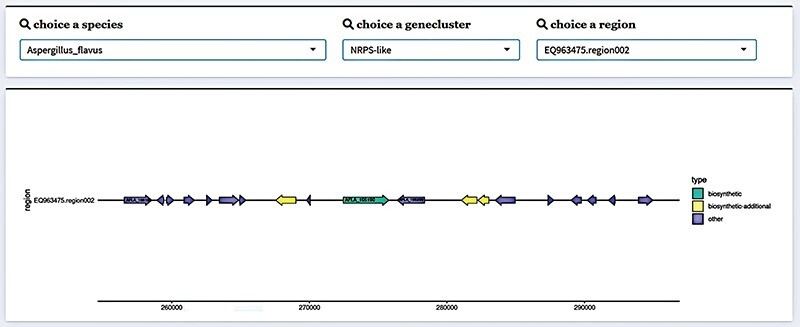
Gene cluster structure information display in FGCD. The selected data can be visually displayed by filtering through the selection box above.

Collecting the gene cluster results from 241 filamentous fungal species identified a total of 7597 gene clusters, which contained 97 159 genes. We visualized the structure of gene clusters using the ‘gggene’ package in R in order to more clearly depict the structure of fungal SM BGCs. Package ‘ggplot2’ in R creates visuals, and ‘gggene’ is an extension package for it. In addition to using functions to draw quickly and simply, it can also draw visuals more freely by superimposing layers. Fungal SM BGC structures are illustrated using this characteristic. First, with the annotation results from antiSMASH, we can extract the location information on the genes in the gene cluster. Next, we can use Python to sort the gene name (molecule), gene name (gene), gene start position (start), gene end position (end) and identify which strand the gene belongs to. Finally, we can use ‘gggene’ to map, where an arrow denotes a gene (Figure 2). In order to provide more accessible gene structure information, this method was used to map the gene cluster structure of SMs from various fungal species. Gene regions, gene cluster information and species information could all be used to select which gene clusters should be shown. In addition, we provided sequence data, encompassing both protein and gene sequences. The page also offered additional gene-related data in the form of a table, including the gene’s location.

**Figure 3.**
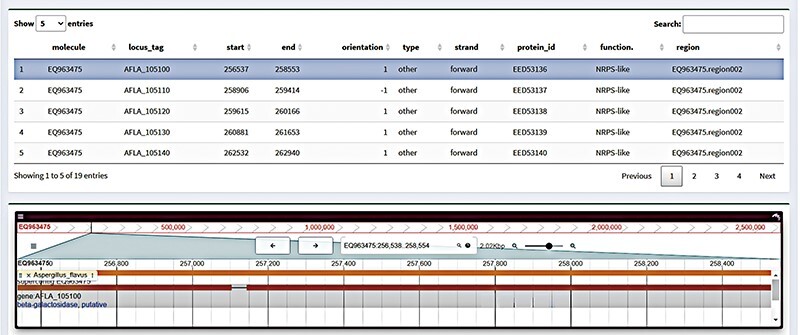
Gene browser interface in FGCD. Click on the table above to display the selected gene in the genome browser.

An online genome browser application can show genomic sequence data and related locations (14). A single-gene genome browser, sometimes referred to as a gene browser, is a genome browser that shows annotation data and particular gene sequences. The genome browser for a single gene typically contains the following data: the gene sequence (which can be found in common formats like FASTA or GenBank), its location on the genome (including chromosome, location and direction) and its annotation data, which includes transcripts, promoters, cleavage sites and regulatory elements. Gene browser section has a search feature that allows one to search for particular genes by location or keyword. Simultaneously, certain chromosome areas or genetic material can be annotated to make subsequent study and analysis easier. We offer a table of data with greater detail on the genes associated with the gene cluster under the ‘gene structure’ presentation. We also improve the interactive operation of the table at the same time. The user can modify the display object of the gene browser by clicking on the associated gene table. Simultaneously, the transcriptomic data display and sequence information (Figure 3) will also offer the same interactive operation.

### Aligning multiple sequences

An analytical technique called multiple sequence alignment is used to compare different biological sequences. Backtracking alignment, local alignment and global alignment are the three multiple sequence alignment techniques used most often. In multiple sequence alignment, users will compare several sequences together and, following alignment, produce a matrix. To assess how similar these sequences are from one another, the matrix keeps track of comparable and dissimilar locations within each sequence. The use of multiple sequence alignment facilitates the investigation of genetic variation among various organisms and their environmental adaptations. Based on the comparative results, users can also carry out additional analysis, such as constructing evolutionary trees and predicting the corresponding protein structures (15).

The NCBI uses BLAST as a tool to compare homologous sequences. The objective is to score the sequence using a certain algorithm and a scoring matrix. The similarity between the two sequences increases with the score. Protein sequences can be compared with other protein sequences using Blastp, and nucleic acids can be compared with other nucleic acid sequences using Blastn. Furthermore, depending on the user’s requirements, tools like Tblastn, Tblastx and Blastx can be employed (16). Furthermore, the localized BLAST can create local databases for comparison, in addition to internet databases, and the local databases can be compared more quickly.

The FGCD database provides a BLAST tool for protein sequences. It is capable of selecting species on its own for multiple sequence alignments. To launch the application, the user types the FASTA format of the alignment sequence in the ‘Enter FASTA sequence(s)’ input box and clicks ‘Start.’ The FGCD BLAST tool offers an easy user experience, being quick to run and simple to operate. The database provides the ability to compare protein sequences multiple times.

### Enrichment analysis based on gene clusters

A bioinformatic method known as enrichment analysis identifies overrepresented or enriched functional categories in a collection of proteins, metabolites or genes. The basic principle is to determine whether a predetermined set of objects (such as genes and proteins) has a statistically significant overlap with functional categories (gene ontologies and pathways) beyond what would be predicted by chance. Making a list of noteworthy entities, such as differentially expressed genes, is the first step in performing enrichment analysis. We call this the query list or input list. Subsequently, the user will require pre-made lists that arrange similar genes according to their function, pathway and ontology. These are derived from databases such as Kyoto Encyclopedia of Genes and Genomes (KEGG) (17) and Gene Ontology (GO) (18) and are referred to as annotation lists. The annotation data from bioinformatic databases are usually employed for enrichment analysis. Their abundance of known information and experimental data makes them an ideal starting point for enrichment analyses. By comparing the datasets of the experimental group with the control group, researchers can use statistical techniques based on these databases to identify enriched functions or pathways. The relationship between various biological processes or pathways and particular traits (reflected in the experimental conditions), such as the molecular mechanism of particular diseases, the adaptive molecular mechanism of specific environments and the biological properties of various tissues, can be better understood by researchers with the aid of enrichment analysis. Furthermore, enrichment analysis can be a useful tool for finding biomarkers in many diseases and biological states by identifying relevant molecular markers or targets (15).

**Figure 4.**
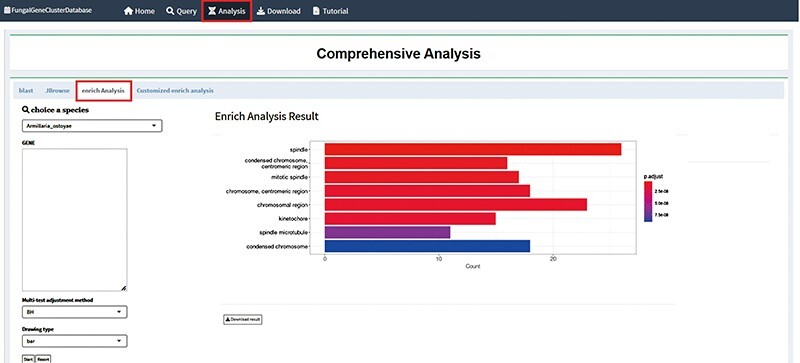
Enrichment analysis interface in FGCD. The obtained gene clusters were used as background information for enrichment analysis.

The fundamental premise of enrichment analysis is that the co-expression of a set of genes results in a biological process (19). Thus, generally, the rate of a biological process is changed under a specific treatment, and the expression of the functionally related genes will change. The objective of enrichment analysis is to investigate the function and significance of genes which regulate life activities using a variety of databases and analytical statistical tools to identify genes exhibiting significant changes in expression (20). When a set of genes is directly annotated, numerous functional nodes are produced; at present, there will be conceptual overlap between these functions. To acquire more meaningful functional information, it is therefore required to filter and screen the functional nodes that have been collected. The most common methods are enrichment analysis with GO and KEGG databases. Transcriptomic analysis essentially reveals variation in gene expression, from which significant KEGG pathways and GO nodes can be identified (21). Ultimately, it can be understood how genes function both inside and outside of organisms. By integrating the ClusterProfiler (https://github.com/YuLab-SMU/clusterProfiler) program as an analytical tool in the background, the FGCD database provides enrichment analysis based on gene clusters (Figure 4) (22). The enrichment analysis allows for downloading of the data and supports two outputs, a bubble chart and a bar chart, for multiple analysis.

### Customized enrichment analysis

With the help of the robust customized background analysis module, customers can customize background information to match their own requirements, enabling a wider range of research applications. All that is needed from the user is the necessary background information file in an acceptable format. After analyzing the data in the file, the platform will present the findings of the analysis in relation to the background data. Based on their actual requirements, users can select background material appropriate for their study objects, improving the accuracy and dependability of data analysis (23).

**Figure 5.**
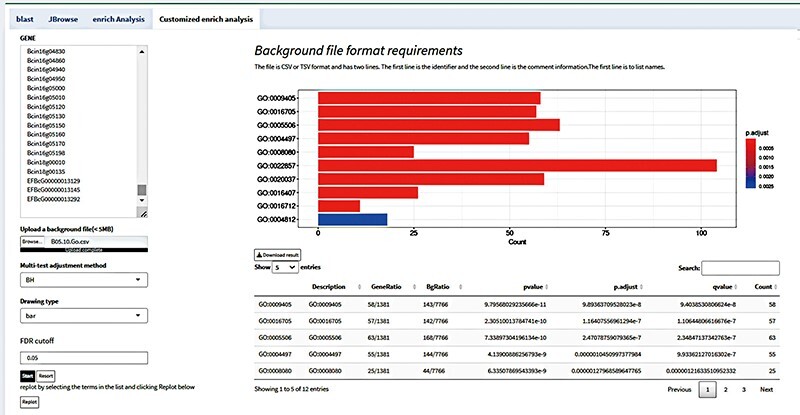
Customize enrichment analysis by uploading background files according to the format.

For instance, transcriptomic analysis was used to identify genes that were differentially expressed using the *Botrytis cinerea* transcriptome data which were generated in our lab. Subsequently, users could access GO background information files pertaining to *Botrytis cinerea* from the BioMart database (https://fungi.ensembl.org/biomart/martview/), and then upload those files to the customized background module for analysis in the format specified (24, 25). Users can obtain the analysis results on which the system will automatically carry out background analysis and provide the results in the form of enrichment analysis. Users can also view the distribution of differentially expressed genes in different pathways as well as a list of differentially expressed genes enriched by a pathway (Figure 5). Furthermore, the module allows the user to focus on a specific pathway. With the help of this tool, users can investigate the intricate relationship between differentially expressed genes and background pathways in greater detail and achieve a more intuitive understanding of how these two entities relate to one another. The customized background analysis module has a wide range of application scenarios and can meet the research needs of different fields. In addition, the high degree of customization possible of the modules also enables it to better adapt to the ever-changing background information needs of different research fields.

### Transcriptomic analysis

Transcription plays a central role in the expression of genetic information. The transcriptome comes from the genome and guides the synthesis of the final product of genome expression, the proteome. With the decline in the cost of second-generation sequencing and the continuous development of bioinformatics technology, transcriptome sequencing has been widely used in all aspects of biological research. Transcriptome sequencing (RNA-Seq) uses high-throughput sequencing technology to measure mRNA sequences, reflect their expression levels and screen out differentially expressed genes. Transcriptome sequencing is divided into four stages. The first stage is to relate the sequenced data back to the reference genome; the second stage is to quantify the expression of genes or transcript abundance; the third stage is to filter and annotate the data; and the fourth stage is to identify which transcripts have undergone significant expression changes. There are many analytical tools available for use in the four stages of transcriptome analysis. Combinations of different analytical tools will produce different analysis processes (26–28). The development of bioinformatics in recent years has also greatly expanded the software available for transcriptomic analysis. Therefore, users need to consider which software to use for particular biological research topics. TrimGalore (23), HISAT2 (29, 30) and Stringtie (31) were used in the current study (32, 33). We have collected transcriptome data for 70 fungal species to date, and we intend to update and expand this data collection when more omics data become available.

### Genome browser

In addition to providing a browser for individual genes in a gene cluster, the FGCD database provides a genome browser for whole genome sequences, also known as JBrowser (Generic Genome Browser) (https://jbrowse.org/jb2/). The genome browser shows clear and simple genome annotation information, which can accurately display gene structure and location information at the genome level, such as gene mapping, exons and introns. In the data display of this genome browser, users can quickly browse the global location and gene spacing of genes to better understand the structure and organization of the genome (34). At the same time, the browser can also display genomic data in a user-friendly way, providing multiple data formats (such as General Feature Format) as well as charts and visualization tools, allowing users to quickly browse and view genomic data. In addition, the browser also provides a variety of interactive features, such as search, location and selection, to support users in better conducting detailed and in-depth genomic data analysis. In general, the genome browser JBrowser of the FGCD database provides users with a clear and simple data display and analysis tool, which enables users to quickly locate and understand the basic organizational structure of the genome and facilitates users to conduct in-depth mining and analysis of genomic data.

## Discussion

Fungal gene clusters refer to a group of adjacent genes that usually encode functionally related proteins. In fungi, identification of these gene clusters is usually regarded as part of the preliminary characterization of genome distribution, and gene clusters are closely associated with the evolution of fungi and their ability to adapt to their environment. In fungi, gene clusters contain genes related to the biosynthesis of bioactive compounds, antibiotics and other adaptive secondary metabolites. These clusters usually consist of more than one gene with specific biological functions, such as enzymes, inducers or regulatory proteins. Therefore, gene clusters are of importance in terms of biosynthesis and biological regulation (35, 36).

The construction of the FGCD fungal secondary metabolite gene cluster database provides a powerful tool for researchers with which to explore such clusters in fungi. The database contains a wealth of secondary metabolite gene cluster information and transcriptomic analysis results. Researchers can use this to screen out and retrieve the required data to explore the biological functions of gene clusters and ultimately to discover new secondary metabolites with potential value. It also facilitates cross-disciplinary and multi-omics data integration and big data analysis. With the help of the FGCD database, researchers can better understand the biological mechanism of secondary metabolite production and explore many aspects of secondary metabolite synthesis, such as the molecular mechanism of the regulation of gene cluster expression, the control strategy of secondary metabolite production, the structure and evolution of gene clusters and the biological function of gene clusters. Taking fungi with potential drug production value as an example, based on the analysis by FGCD, it is easier to find the direction and strategy of drug-development-targeted gene clusters. At the same time, the deep mining of fungal gene clusters associated with secondary metabolite biosynthesis is expected to promote the development of novel strategies for studying metabolites for agricultural and environmental protection.

In addition, FGCD also provides data visualization and analysis tools. For example, users can visualize the location of gene clusters and their distribution on the genome through the use of a genome browser. At the same time, FGCD also integrates BLAST tools to facilitate users in performing homologous alignment of specific sequences and further studying the function and evolutionary history of clustered sequences. In addition, for analysis of the biological significance and function of gene clusters, FGCD also provides users with enrichment analysis tools to help researchers identify biological functions and pathways related to gene clusters and provide strong support for subsequent experimental design and further exploration. Currently, the FGCD database has included information on the secondary metabolite gene clusters of 241 filamentous fungal species, including a total of 7597 gene clusters and 97 159 genes. The establishment and operation of the FGCD database enables researchers to study the gene clusters of fungal secondary metabolites more conveniently and efficiently. Through in-depth exploration of the biological significance and function of gene clusters, it provides important data resources and technical support for the development and application of fungal secondary metabolites. At the same time, FGCD will continue to be updated, expanded and improved and strive to cover more gene cluster information from fungal species, providing researchers with more comprehensive and accurate data resources and analytical tools.

## Conclusion

To facilitate the identification, analysis and display of fungal secondary metabolite gene clusters, we constructed a database of fungal gene clusters controlling SM (FGCD) as described in this study. Currently, the FGCD has a large dataset from 241 fungal species, which includes 7597 gene clusters in total and 97 159 genes. We aim to expand the database to cover more species in the future and will keep updating the database. The key motivations for creating this database include: (i) accurate identification and annotation of the secondary metabolite biosynthesis pathways encoded by each gene cluster, providing insights into metabolite structure and function; (ii) integration of fungal secondary metabolite gene cluster data from multiple sources into one centralized, easily accessible database for straightforward comparative analysis; (iii) the saving of significant time and effort by researchers by avoiding redundant bioinformatic analysis and enabling rapid data queries; (iv) direct presentation of gene cluster architecture and visualization, rather than raw sequence data, allowing users to immediately view gene organization; and (v) depiction of gene cluster structures, such as size, gene content and genomic location through user-friendly maps. In addition, the utility and scope of the FGCD will expand through ongoing updates with newly sequenced fungal genomes and RNA-Seq data. The FGCD serves as a valuable open access reference tool and repository for the fungal research community investigating this biotechnologically and ecologically important class of gene clusters.

## Data Availability

Genome data were obtained from the Ensembl Fungi database (https://fungi.ensembl.org/index.html). Results Data This database is available for download.

## References

[1] Katz L. and Baltz R.H. (2016) Natural product discovery: past, present, and future. *J. Ind. Microbiol. Biotechnol*., 43, 155–176. 26739136 10.1007/s10295-015-1723-5

[2] Chakraborty P. (2022) Gene cluster from plant to microbes: their role in genome architecture, organism’s development, specialized metabolism and drug discovery. *Biochimie*, 193, 1–15. 34890733 10.1016/j.biochi.2021.12.001

[3] Brun T. , Rabuske J.E., Confortin T.C. et al. (2022) Weed control by metabolites produced from Diaporthe schini. *Environ. Technol*., 43, 139–148. 32510281 10.1080/09593330.2020.1780477

[4] Xu Z. , Park T.J. and Cao H. (2023) Advances in mining and expressing microbial biosynthetic gene clusters. *Crit. Rev. Microbiol*., 49, 18–37. 35166616 10.1080/1040841X.2022.2036099

[5] Kang H.S. and Kim E.S. (2021) Recent advances in heterologous expression of natural product biosynthetic gene clusters in Streptomyces hosts. *Curr. Opin. Biotechnol*., 69, 118–127. 33445072 10.1016/j.copbio.2020.12.016

[6] Trapnell C. , Hendrickson D.G., Sauvageau M. et al. (2013) Differential analysis of gene regulation at transcript resolution with RNA-seq. *Nat. Biotechnol*., 31, 46–53. 23222703 10.1038/nbt.2450 PMC3869392

[7] Kayrouz C.M. , Huang J., Hauser N. et al. (2022) Biosynthesis of selenium-containing small molecules in diverse microorganisms. *Nature*, 610, 199–204. 36071162 10.1038/s41586-022-05174-2

[8] Dean R.A. (2007) Fungal gene clusters. *Nat. Biotechnol*., 25, 67. 10.1038/nbt0107-67 17211403

[9] Barrett T. , Wilhite S.E., Ledoux P. et al. (2013) NCBI GEO: archive for functional genomics data sets—update. *Nucleic Acids Res*., 41, D991–D995. 23193258 10.1093/nar/gks1193 PMC3531084

[10] Blin K. , Shaw S., Kloosterman A.M. et al. (2021) antiSMASH 6.0: improving cluster detection and comparison capabilities. *Nucleic Acids Res*., 49, W29–W35. 33978755 10.1093/nar/gkab335 PMC8262755

[11] Blin K. , Medema M.H., Kottmann R. et al. (2017) The antiSMASH database, a comprehensive database of microbial secondary metabolite biosynthetic gene clusters. *Nucleic Acids Res*., 45, D555–D559. 27924032 10.1093/nar/gkw960 PMC5210647

[12] Giorgi F.M. , Ceraolo C. and Mercatelli D. (2022) The R Language: an engine for bioinformatics and data science. *Life*, 12, 648. 10.3390/life12050648 PMC9148156 35629316

[13] Jia L. , Yao W., Jiang Y. et al. (2022) Development of interactive biological web applications with R/Shiny. *Brief. Bioinform*., 23, bbab415. 10.1093/bib/bbab415 34642739

[14] Skinner M.E. , Uzilov A.V., Stein L.D. et al. (2009) JBrowse: a next-generation genome browser. *Genome Res*., 19, 1630–1638. 19570905 10.1101/gr.094607.109 PMC2752129

[15] Chen X. and Tompa M. (2010) Comparative assessment of methods for aligning multiple genome sequences. *Nat. Biotechnol*., 28, 567–572. 20495551 10.1038/nbt.1637

[16] McGinnis S. and Madden T.L. (2004) BLAST: at the core of a powerful and diverse set of sequence analysis tools. *Nucleic Acids Res*., 32, W20–W25. 15215342 10.1093/nar/gkh435 PMC441573

[17] Kanehisa M. , Furumichi M., Tanabe M. et al. (2017) KEGG: new perspectives on genomes, pathways, diseases and drugs. *Nucleic Acids Res*., 45, D353–D361. 27899662 10.1093/nar/gkw1092 PMC5210567

[18] Gene Ontology Consortium . (2015) Gene ontology consortium: going forward. *Nucleic Acids Res*., 43, D1049–D1056. 25428369 10.1093/nar/gku1179 PMC4383973

[19] Tian T. , Liu Y., Yan H. et al. (2017) AgriGO v2.0: a GO analysis toolkit for the agricultural community, 2017 update. *Nucleic Acids Res*., 45, W122–W129. 28472432 10.1093/nar/gkx382 PMC5793732

[20] Huang D.W. , Sherman B.T. and Lempicki R.A. (2009) Bioinformatics enrichment tools: paths toward the comprehensive functional analysis of large gene lists. *Nucleic Acids Res*., 37, 1–13. 19033363 10.1093/nar/gkn923 PMC2615629

[21] Yu G. (2020) Gene ontology semantic similarity analysis using GOSemSim. *Methods Mol. Biol*., 2117, 207–215. 31960380 10.1007/978-1-0716-0301-7_11

[22] Wu T. , Hu E., Xu S. et al. (2021) ClusterProfiler 4.0: a universal enrichment tool for interpreting omics data. *Innovation*, 2, 100141. 10.1016/j.xinn.2021.100141 PMC8454663 34557778

[23] Yu G. , Wang L.G., Yan G.R. et al. (2015) DOSE: an R/Bioconductor package for disease ontology semantic and enrichment analysis. *Bioinformatics*, 31, 608–609. 25677125 10.1093/bioinformatics/btu684

[24] Haider S. , Ballester B., Smedley D. et al. (2009) BioMart central portal—unified access to biological data. *Nucleic Acids Res*., 37, W23–W27. 19420058 10.1093/nar/gkp265 PMC2703988

[25] Smedley D. , Haider S., Ballester B. et al. (2009) BioMart—biological queries made easy. *BMC Genom*., 10, 22. 10.1186/1471-2164-10-22 PMC2649164 19144180

[26] Kim D. , Pertea G., Trapnell C. et al. (2013) TopHat2: accurate alignment of transcriptomes in the presence of insertions, deletions and gene fusions. *Genome Biol*., 14, R36. 10.1186/gb-2013-14-4-r36 PMC4053844 23618408

[27] Roberts A. , Trapnell C., Donaghey J. et al. (2011) Improving RNA-Seq expression estimates by correcting for fragment bias. *Genome Biol*., 12, R22. 10.1186/gb-2011-12-3-r22 PMC3129672 21410973

[28] Roberts A. , Pimentel H., Trapnell C. et al. (2011) Identification of novel transcripts in annotated genomes using RNA-Seq. *Bioinformatics*, 27, 2325–2329. 21697122 10.1093/bioinformatics/btr355

[29] Kim D. , Paggi J.M., Park C. et al. (2019) Graph-based genome alignment and genotyping with HISAT2 and HISAT-genotype. *Nat. Biotechnol*., 37, 907–915. 31375807 10.1038/s41587-019-0201-4 PMC7605509

[30] Zhang Y. , Park C., Bennett C. et al. (2021) Rapid and accurate alignment of nucleotide conversion sequencing reads with HISAT-3N. *Genome Res*., 31, 1290–1295. 34103331 10.1101/gr.275193.120 PMC8256862

[31] Pertea M. , Pertea G.M., Antonescu C.M. et al. (2015) StringTie enables improved reconstruction of a transcriptome from RNA-seq reads. *Nat. Biotechnol*., 33, 290–295. 25690850 10.1038/nbt.3122 PMC4643835

[32] Li H. , Handsaker B., Wysoker A. et al. (2009) The Sequence Alignment/Map format and SAMtools. *Bioinformatics*, 25, 2078–2079. 19505943 10.1093/bioinformatics/btp352 PMC2723002

[33] Danecek P. , Bonfield J.K., Liddle J. et al. (2021) Twelve years of SAMtools and BCFtools. *GigaScience*, 10, giab008. 10.1093/gigascience/giab008 PMC7931819 33590861

[34] Hershberg E.A. , Stevens G., Diesh C. et al. (2021) JBrowseR: an R interface to the JBrowse 2 genome browser. *Bioinformatics*, 37, 3914–3915. 34196689 10.1093/bioinformatics/btab459 PMC8570803

[35] Rokas A. , Mead M.E., Steenwyk J.L. et al. (2020) Biosynthetic gene clusters and the evolution of fungal chemo diversity. *Nat. Prod. Rep*., 37, 868–878. 31898704 10.1039/c9np00045c PMC7332410

[36] Slot J.C. (2017) Fungal gene cluster diversity and evolution. *Adv. Genet*., 100, 141–178. 29153399 10.1016/bs.adgen.2017.09.005

